# The lowering effect of Gum Arabic on hyperlipidemia in Sudanese patients

**DOI:** 10.3389/fphys.2015.00160

**Published:** 2015-05-18

**Authors:** Rima E. Mohamed, Mohammed O. Gadour, Ishag Adam

**Affiliations:** ^1^Faculty of Medicine, Omdurman UniversityOmdurman, Sudan; ^2^Faculty of Medicine, University of KhartoumKhartoum, Sudan

**Keywords:** hyperlipidemia, hypercholestrolemia, Gum Arabic, Sudan

## Abstract

Hyperlipidemia especially low density lipoprotein cholesterol (LDL-C) is a major risk factor for developing ischemic heart disease. Soluble dietary fiber has lipid lowering characteristics. Gum Arabic (GA) is 95% soluble fiber calculated on dry bases. The beneficial effect of GA on lipid profile needs further verification. A case–control study was conducted at Omdurman Hospital, Sudan to assess the effect of G A on serum lipids in patients with hyperlipidemia. Cases received a 20 mg tablet of atorvastatin /day plus 30 mg of GA for 4 weeks while the controls received atorvastatin only. Levels of lipids in serum were assessed according to conventional methods before and 1 month after the trial. There is no significant difference in the basic characteristics between the study and the control groups (55 patients in each arm of the study). While there was no significant difference in the levels of HDL, there was a significant reduction of the total cholesterol (25.9 vs. 7.8%, *P* < 0.001), triglyceride (38.2 vs. 2.9%, *P* < 0.001), and LDL (30.8 vs. 8.1%, *P* < 0.001) before and after the intervention in the study compared to the controls groups.

## Introduction

It has been shown that elevated cholesterol level -especially low density lipoprotein cholesterol (LDL-C)–is a major risk factor for coronary heart disease (CHD) which is a leading cause of death worldwide. Soluble dietary fiber has a lipid-lowering property (Pekkanen et al., 1990). The Adult Treatment Panel III (ATP III) of the National Cholesterol Education Program (NCEP) issued an evidence-based set of guidelines on cholesterol management in which they encouraged use of viscous (soluble) fiber (e.g., oats, guar, pectin, and psyllium) as therapeutic dietary options to enhance lowering of LDL cholesterol in primary and secondary prevention of CHD(NCEP, 2002) Gum Arabic (GA) which is mixture of polysaccharides, oligosaccharides and glycoproteins is exudates of Acacia Senegal/seyal trees (Anderson and Stoddart, 1966; Goodrum et al., 2000). GA is defined by the FAO/WHO Joint Expert Committee as an exudation obtained from the stems of Acacia Senegal or closely related other species of Acacia (FAO/WHO, 1969; FAO, 1990). It is water soluble; therefore it is used as an emulsifier, thickening substance and flavor stabilizer in many pharmaceutical and food industries (Dziezak, 1991). Interestingly, since 1969 GA was evaluated for acceptable daily intake for man by the Joint FAO/WHO Expert Committee on Food Additives since 1969 (FAO/WHO, 1969; FAO, 1990). GA is indigestible to humans, but following its fermentation in the colon short-chain fatty acids are produced that can lead a lot of benefits such as prebiotic effect, increases in Bifidobacteria, Lactobacteria, and Bacteriodes reflecting a prebiotic effect (Calame et al., 2008; Phillips et al., 2008; Phillips and Phillips, 2011), anticarcinogenic effect and anti-oxidant effect (Sharma, 1985; Al-Majed et al., 2002; Ali et al., 2003; Nasir et al., 2010).

Other effects of GA include reduction in plasma cholesterol level in animals and humans (Sharma, 1985). Lowering effect of GA on lipid profile that has been mentioned in some studies needs further verification and more research is necessary (Kelley and Tsai, 1978; Ross et al., 1983; Sharma, 1985; Mee and Gee, 1997). The aim of this study was to determine the effects of GA ingestion on lipids profile among adults Sudanese patients with hyperlipidemia.

## Materials and methods

### Patients

A case–control was conducted at Omdurman Hospital Sudan during the period of June–December 2012 where patients with hyperlipidemia (total cholesterol > 200 mg/dl, LDL > 100 mg/dl), HDL < 40 mg/dl, triglyceride > 150 mg/dl were enrolled to the study. A sample size of 55 subjects per group was calculated to detect a significant reduction in lipid profile 15–25%, with a two-sided 5% significance level and a power of 80% (Whitley and Ball, 2002).

The patients with newly discovered hyperlipidemia were enrolled (if they were not on lipid lowering agent for at least 1 month before starting the study. Patients who had familial hyperlipidemia, premature CHD, pregnant, and lactating ladies were excluded. Using computer generated number and sealed envelopes patients were assigned to the study group received atorvastatin tablet, 20 mg plus 30 mg of GA which was a natural gum provided in a powder form by the expertise “Dar Savanna Ltd. Khartoum, Sudan” that provided it for the research before (Babiker et al., 2012) and control group received statin only. Both statin and GA were used at bedtime. The socio-demographic, medical history was taken from each patient using questionnaire. The control groups were advised not to take GA by themselves (it is widely used in Sudan). The diet and exercise were kept under control in both the study and controls groups. Then 5 ml of venous blood was taken from each respondent to measure lipid profile (total, LDL and HDL cholesterol and triglycerides) using a multichannel chemistry auto analyzer Lipid profiles, were determined for each participant twice; at baseline of the study and after 4 weeks. Compliance was monitored by weekly contact with the subjects by phone.

### Statistics

Data were entered using SPSS for Windows V.16.0 (SPSS Inc., Chicago, IL, USA. The paired *t*-test was used for analysis of pre and post intervention levels of the lipid profile. Mean (SD) and proportion were compared between the study and control groups using independent sample *t*-test and X^2^, respectively. A *p*-value of less than 0.05 was regarded statistically significant.

### Ethics

The study received ethical clearance from the board of ethical committee of Ministry of Health Khartoum State, Sudan. Confidentiality and privacy of respondents was assured. All responses were kept anonymous. Coding (Identification numbers) was used to identify the data collection form.

## Results

One hundred and twenty patients with hyperlipidemia who met the inclusion and exclusion criteria were enrolled to the study. Out of these 120, 110 completed the study through the end of the 4-weeks treatment period. Ten (5 in each group) participants dropped due to loss of follow-up. There was no significant difference in the mean (SD) of the age [54.7(10.4) vs. 55.2(11.3) years, *P* = 0.809], body mass index [29.7 (7.2) vs. 29.1(6.5) kg/cm^2^, *P* = 0.454] and the number (%) of female [31(56.4%) vs. 30 (54.5%), *p* > 0.999] between the study and the control groups.

**Figure 1:**
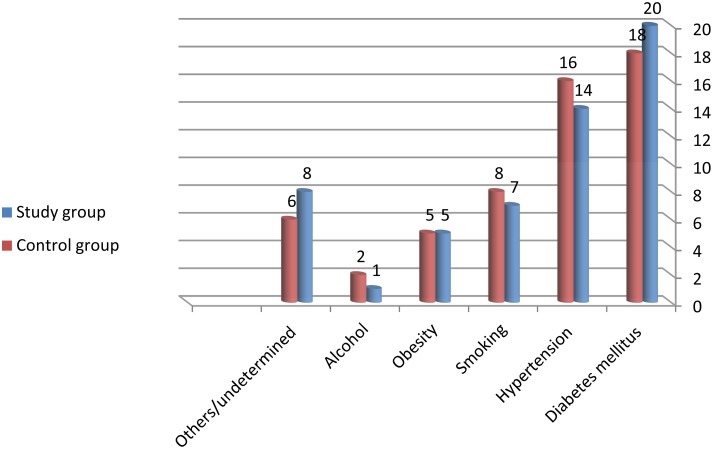
**Different causes of the hyperlipidemia in the study and control group**.

Different causes of hyperlipidemia were observed and these were not different between the study and control groups, Figure 1.

**Figure 2:**
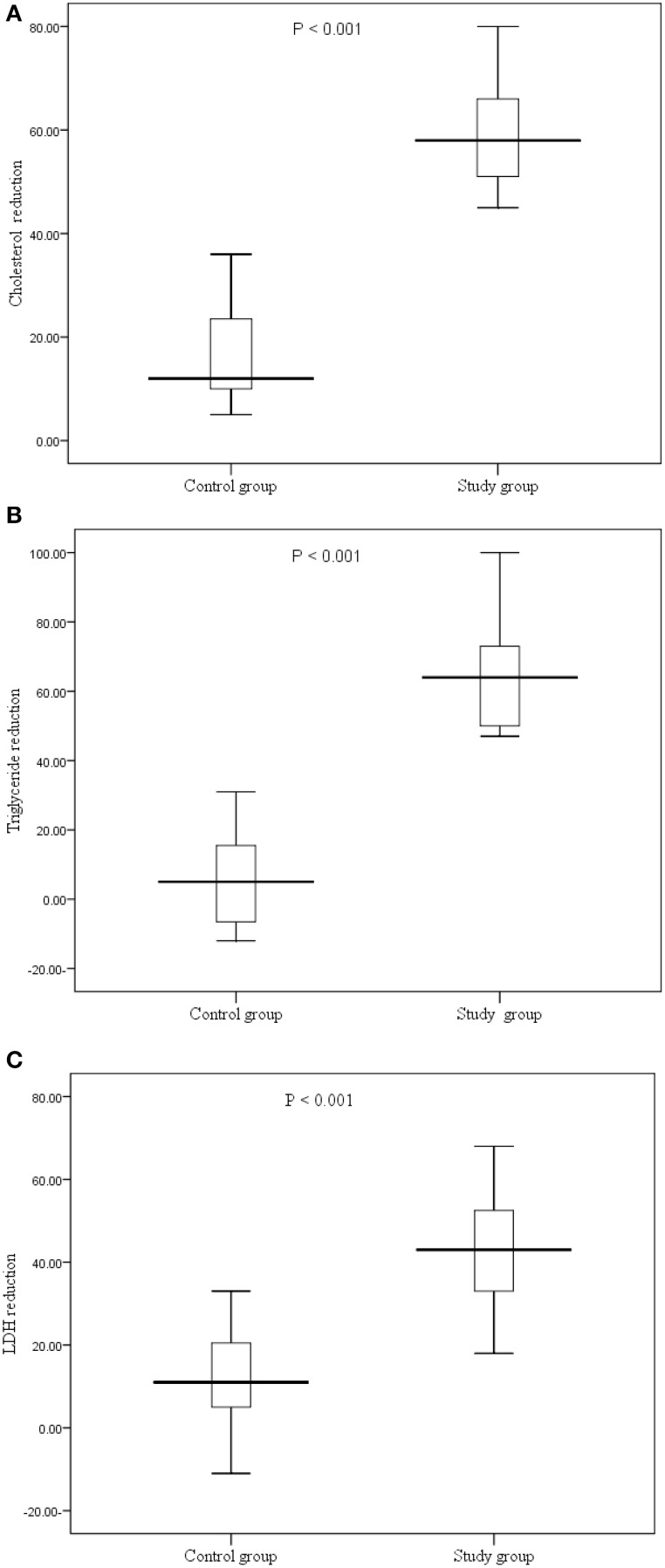
**(A–C)** Reduction of the lipids (cholesterol, triglyceride and LDL profile in the study and control groups before and after the trial.

While there was no significant difference in the levels of HDL, there was a significant reduction of the total cholesterol (25.9 vs. 7.8%, *P* < 0.001), triglyceride (38.2 vs. 2.9%, *P* < 0.001) and LDL (30.8 vs. 8.1%, *P* < 0.001) in the study compared to the controls groups, Table 1, and Figure 2.

**Table 1:** **Changes of the lipids profile in the study and control groups before and after the trial**.

**Variable**		**Mean (SD)**	**Mean (%) difference**	***P***	***P***
Cholesterol (study group)	Before	226.4 (9.0)	58.7 (25.9)	< 0.001	< 0.001
	After	167.6 (3.6)			
Cholesterol (control group)	Before	225.9 (8.7)	17.8 (7.8)	< 0.001	
	After	208.0 (6.4)			
Triglyceride (study group)	Before	171.6 (13.3)	65.5 (38.2)	< 0.001	< 0.001
	After	106.0 (7.1)			
Triglyceride (control group)	Before	172.6 (13.0)	5.1 (2.9)	0.005	
	After	167.4 (1.7)			
HDL (study group)	Before	45.9 (4.6)	1.5 (3.3)	0.172	0.945
	After	44.4 (6.6)			
HDL (control group)	Before	46.0 (4.6)	1.6 (3.6)	0.136	
	After	44.4 (6.6)			
LDL (study group)	Before	141.2 (13.0)	43.6 (30.8)	< 0.001	< 0.001
	After	97.6 (4.5)			
LDL (control group)	Before	142.5 (11.7)	11.5 (8.1)	< 0.001	
	After	130.9 (1.3)			

## Discussion

The main finding of the current study is the lowering effect of the GA on the lipid levels (with exception of HDL) in patients with hyperlipidemia. This goes with the previous findings from other studies that have reported lipid-lowering effects of soluble dietary fiber (Ross et al., 1983; Judd and Truswell, 1985; Sharma, 1985; Anderson et al., 1991; Glore et al., 1994). However, Haskell et al. (1992) showed that a low-viscosity acacia gum at a dose of 15 g/d had no effect on the lipid profile compared to placebo. Likewise, Davidson et al. (1998) observations did not support the hypocholesterolemic effect of the GA/pectin. The variable lipid lowering effects of GA might be related to the dose used, the chemical constituents of GA that can vary with its source or may be due to genetic and personal differences (Judd and Truswell, 1985; Islam et al., 1997; Verbeken et al., 2003). It has been reported that dietary fibers including GA bind bile acids and diminish their absorption in the terminal ileum (Moundras et al., 1994). Then in the large intestine, breakdown of GA releases the sequestered bile acids and the acidic pH generated during the fermentation process renders them insoluble and promotes their excretion in stool (Moundras et al., 1994). This process reduces the lipid pool in the body and causes decreased fat digestion and absorption. Likewise, the liver formation of new bile acids requires cholesterol. Thus, prolonged ingestion of GA may lead to reduction in cholesterol level in plasma. Interestingly a more a recent proposed mechanism by which viscous dietary fibers can reduce adiposity is increased mitochondrial biogenesis and fatty acid oxidation by skeletal muscles (Islam et al., 2012).

In summary, the current study showed a statistically significant reduction in total cholesterol, triglyceride and LDL with in the study group (GA) compared to the control group. Prospective studies with larger number of participants and longer duration together with the evaluation of the effect of GA alone on lipid profile are needed.

### Conflict of interest statement

The authors declare that the research was conducted in the absence of any commercial or financial relationships that could be construed as a potential conflict of interest.
